# Evaluation of the Therapeutic Effect of Adjuvant Transcatheter Arterial Chemoembolization Based on Ki67 After Hepatocellular Carcinoma Surgery

**DOI:** 10.3389/fonc.2021.605234

**Published:** 2021-02-25

**Authors:** Yu-Fei Zhao, Xiu Xiong, Kai Chen, Wei Tang, Xu Yang, Zheng-Rong Shi

**Affiliations:** Department of Hepatobiliary Surgery, The First Affiliated Hospital of Chongqing Medical University, Chongqing, China

**Keywords:** hepatocellular carcinoma (HCC), ki67, postoperative adjuvant transcatheter arterial chemoembolization (PA-TACE), propensity score matching (PSM), prognosis

## Abstract

**Background and aims:**

This study aimed to determine the relationship between Ki67 expression and the efficacy of postoperative adjuvant transcatheter arterial chemoembolization (PA-TACE) in patients with hepatocellular carcinoma.

**Methods:**

The Kaplan-Meier method was used to analyze the recurrence-free survival (RFS) and overall survival (OS) rates between the sub-groups in the ki67 low expression group and the ki67 high expression group and analyze the relationship between the expression of Ki67 and the efficacy of TACE.

**Results:**

After PSM, there was no significant difference in the RFS and OS between the surgery + TACE and surgery subgroups after 1, 2, or 3 years (RFS: 63.9%, 55.6%, and 42.9% vs. 83.3%, 63.9%, and 55.6%, respectively, P = 0.279; OS: 91.7%, 83.3%, and 74.3% vs. 91.7%, 88.9%, and 71.4%, respectively, P = 0.890) in the Ki67 low-expression group. The RFS and OS were higher in the surgery + TACE subgroup than the surgery subgroup after 1, 2, and 3 years (RFS: 80.0%, 77.5%, and 69.2% vs. 53.5%, 39.5%, and 32.6%, respectively, P<0.001; OS: 97.5%, 85.0%, and 79.5% vs. 79.1%, 48.8%, and 42.9%, respectively, P = 0.001) in the Ki67 high expression group. The RFS was higher in the Ki67 high-expression subgroup than the low-expression subgroup after 1, 2, and 3 years, and OS had no significant difference (RFS: 80.0%, 79.5%, and 69.2% vs. 67.4%, 56.5%, and 46.7%, respectively, P = 0.035; OS: 97.5%, 85.0%, and 79.5% vs. 93.5%, 82.6%, and 75.6%, respectively, P = 0.665) in the surgery + TACE group.

**Conclusions:**

For patients with hepatocellular carcinoma and high expression of Ki67 (Ki67≥20%), adjuvant hepatic artery chemoembolization after radical liver tumor resection effectively reduced the probability of tumor recurrence after surgery and prolonged the OS of patients. High Ki67 expression during the post-operative follow-up evaluation of hepatocellular carcinoma patients is an indicator for adjuvant TACE therapy.

## Introduction

A radical liver resection is currently the most effective way to treat patients with hepatocellular carcinoma (HCC) (1), and studies have shown that the recurrence rate of patients undergoing radical liver resection alone is approximately 70% 5 years after surgery, while the 5-year survival rate is decreased by 24% in patients with a HCC recurrence compared to patients without a recurrence (2). Indeed, the median survival time is decreased by 54 months in patients with a HCC recurrent (2). Postoperative adjuvant treatment can effectively delay tumor recurrence and prolong the survival of patients. Specifically, transcatheter arterial chemoembolization (TACE) is currently the most widely used adjuvant treatment program for patients with HCC (3). A large number of studies in China and abroad have confirmed that for patients with tumors >5 cm in diameter, multinodular tumors, or MVI-positive HCC, post-operative adjuvant transcatheter arterial chemoembolization (PA-TACE) can effectively improve the overall survival (OS) and disease-free survival (DFS) (4); however, a clear standard for patients who will benefit from PA-TACE has not been established. Therefore, further research is needed to determine the selection criteria for patients who are candidates for TACE after radical liver resection. The development of a precise standard will facilitate clinical decision-making and treat patients in a timely fashion, thus improving the postoperative tumor recurrence and survival rates.

Ki67 is an antigen that reflects the active status of cell proliferation and is closely related to the prognosis of malignant tumors (5). Studies have shown that Ki67 is an independent risk factor for disease-free survival (DFS) and overall survival (OS) in HCC patients (6). Studies have shown that the expression of Ki67 in HCC patients is of considerable value in predicting the postoperative recurrence of HCC (7). The liver cancer blood supply is mainly provided by the hepatic artery, and TACE embolizes the main blood vessel supplying the tumor, resulting in ischemia and necrosis of the tumor tissue in the embolized area. TACE commonly uses chemotherapy drugs, such as oxaliplatin (8) and irinotecan (9), and epirubicin (10), which all have the main effect of inhibiting tumor cell proliferation and inhibiting DNA replication. We speculate that HCC patients with high expression of Ki67 have more active cell proliferation, and the therapeutic effect of postoperative adjuvant TACE will be better. If so, this approach can serve as a guide to determine if PA-TACE should be performed. At present, there are few studies involving Ki67 and PA-TACE in China and abroad (11). The current study will determine the relationship between the expression of Ki67 and the prognosis of patients undergoing radical liver resection and PA-TACE and to guide the use of adjuvant therapy.

## Materials and Methods

### General Information

This study was performed under a human investigational protocol that was approved and monitored by the Institutional Review Board of The First Affiliated Hospital of Chongqing Medical University (the ethical approval number:2019-021). The Ethics Committee also approved the retrospective analysis of existing patient data without informed consent because of the low risk for breaching confidentiality. A retrospective analysis was conducted using the clinical data of patients with liver cancer who underwent radical liver resection in the Department of Hepatobiliary Surgery at the First Affiliated Hospital of Chongqing Medical University from January 2013 to June 2017. The inclusion criteria were as follows: 1) preoperative liver function Child-Pugh score A/B, and liver reserve function indicating sufficient residual liver volume; 2) radical liver tumor resection, and postoperative medical examination confirming that no cancer cells are involved in the resection margin; 3) hepatocellular carcinoma confirmed by postoperative medical examination and immunohistochemical analysis; 4) no portal vein or other large blood vessel invasion or distant metastasis; and 5) patients in the interventional group who received 1–2 TACE treatments after surgery. The exclusion criteria were as follows: 1) tumor recurrence demonstrated within 2 month after surgery or during TACE therapy; 2) co-existing tumors; 3) adjuvant treatments other than TACE performed during the interval between the first diagnosis of recurrence or metastasis after surgery; and 4) loss to follow-up in <1 year. A total of 180 patients with HCC after liver resection were enrolled; 94 patients underwent radical liver resection and 86 patients received adjuvant TACE treatment after liver resection. The clinical data (gender, age, hepatitis B history, co-existing liver cirrhosis, preoperative liver function, and preoperative AFP level) and related data (maximum diameter of lesions, MVI, the degree of tumor differentiation and expression of Ki67) were collected. The preoperative liver function was determined by the Child-Pugh score. The number and maximum diameter of the lesions in the surgically-resected specimens were measured. MVI, Liver cirrhosis, the degree of tumor differentiation and the Ki67 level was determined by a pathologist in the Pathology Department.

Tissue samples from non-necrotic areas were selected from HCC specimens obtained by surgery and fixed in 10% paraformaldehyde for 24 hours. They were then treated with tissue processor, dehydrated and embedded in paraffin. After sections, they were washed with phosphate buffer saline (PBS) for 5 min and added with 3% H_2_O_2_ at room temperature for 10 min. A primary antibodies raised against Ki67 was then added for immunohistochemical detection. Results were interpreted from active cancer tissues and phosphate buffer was used as negative control. Several high-magnification microscopic fields were observed randomly, the percentage of positive cells was calculated and the average value was calculated. All tissue specimens were examined and reported by a qualified pathologist in the Department of Pathology of our hospital. (Interpretation criteria: Ki67 positive cells with thick brown yellow particles in the nucleus were randomly selected from 5 different high-power fields to calculate the percentage of the number of Ki67 positive cells in the total number of observed cells).

### Therapeutic Conditions

Preoperative liver function for all patients was rated as grade A/B and the imaging examinations showed no large vessel invasion or distant metastasis. Liver reserve function suggested that the residual liver volume was sufficient postoperatively. When the tumor was confined locally or occupied one-half of the liver, an anatomic liver resection was performed. When multiple tumors occupied the two half-livers, tumor enucleations were performed separately. All postoperative adjuvant TACE patients were included in a multidisciplinary discussion (including hepatobiliary and pancreatic surgery, oncology, pathology, and radiology), and the patients at high risk of recurrences, such as maximum diameter of lesions >5 cm, MVI>M1 and less differentiated tumor after curative hepatectomy, TACE was indicated. Within 1 month after liver resection, the Seldinger technique was used to puncture the catheter through the femoral artery, and chemotherapy embolization was performed with injection of oxaliplatin, irinotecan, pirarubicin, or epirubicin and lipiodol in the proper hepatic artery. The dosage was determined based on body surface area and liver function. A total of 1–2 TACE treatments were given, with an interval of at least 3 weeks, and liver function was assessed before surgery to confirm the ability to withstand interventional therapy. None of the patients with HCC enrolled in the group received other types of adjuvant therapy, such as targeted therapy, immunotherapy, or absolute alcohol injection, from the time of surgery to the first diagnosis of a recurrence or metastasis.

### Follow-Up

All patients were followed regularly in the outpatient clinics (every 3 months for 1 year after surgery, then every 6 months for 1 year). The outpatient follow-up evaluations included liver and kidney function tests, HBV-DNA quantification, tumor marker profile, abdominal color Doppler ultrasound or abdominal enhanced CT, and a chest CT scan. The endpoint of follow-up was June 20, 2020. The median duration of follow-up was 47 months (95% CI: 43.2–50.7 months). The diagnostic criteria for tumor recurrence were consistent with the initial diagnostic criteria for HCC. The follow-up endpoint of this study was tumor recurrence or metastasis, as indicated by imaging.

### Statistics

SPSS 22.0 software was used for statistical analysis. The endpoint of the study was overall survival (OS). The OS was the time from the first operation to the death from any causes. Propensity score matching (PSM) was used to reduce the bias in clinical and medical examination data between the groups. The two groups were matched according to the 1:1 nearest neighbor matching method, and the standard deviation was <0.2. A χ2 test was used for comparisons between groups. The Kaplan-Meier method was used for survival analysis, and a log-rank test was used for comparisons between groups. The Cox risk ratio model was used to analyze independent risk factors affecting prognosis. A P < 0.05 was considered statistically significant.

## Results

### Basic Information of Patients

From January 2013 to June 2017, a total of 180 patients with HCC underwent surgical treatment; 94 patients underwent radical liver resection alone (operating group) and 86 patients received adjuvant TACE treatment after liver resection (operation + TACE group). There was no statistical difference between the two groups with respect to clinical data (age, sex, hepatitis B history, preoperative liver function, and pre-operative AFP level) and disease examination-related data (number of lesions, maximum diameter of lesions, and expression of Ki67). The proportion of patients with liver cirrhosis in the pre-PSM surgery group was higher. After propensity matching eliminated the difference variables, 83 pairs of HCC patients were assigned. There was no statistical difference between the two covariates (P value >0.05, **Table 1**). The patients were divided into the Ki67 low- and high-expression subgroups. There was no statistical difference between the two groups with respect to clinical data and disease examination-related data (P value >0.05, **Table 2**).

**Table 1:** Basic information of all patients in terms of treatment options[cases(%)].

Clinical data	Pre-PSM			After PSM		
	Operating group(n=94)	operation + TACE group(n=86)	P	Operating group(n=83)	operation + TACE group(n=83)	P
Age(>55)	51(54.3)	35(40.7)	0.069	43(51.8)	33(39.8)	0.119
Gender(male)	83(88.3)	74(86.0)	0.651	72(86.7)	71(85.5)	0.822
Hepatitis B history	81(86.2)	78(90.7)	0.345	73(88.0)	75(90.4)	0.618
Liver cirrhosis	66(70.2)	48(55.8)	0.045	55(66.3)	48(57.8)	0.263
Pre-operative liver function(A)	93(98.9)	83(96.5)	0.270	82(98.8)	80(96.4)	0.311
AFP(>200 μg/liter)	28(29.8)	26(30.2)	0.948	24(28.9)	24(28.9)	1.000
Number of lesions(single)	82(87.2)	66(76.7)	0.066	71(85.5)	65(78.3)	0.226
Maximum diameter of lesions(>5 cm)	50(53.2)	44(51.2)	0.785	40(48.2)	43(51.8)	0.641
Ki67 (high expression)	43(45.7)	40(46.5)	0.918	37(44.6)	38(45.8)	0.876

The proportion of patients with liver cirrhosis in the pre-PSM surgery group was higher.

**Table 2:** Basic information of all patients in the expression of Ki67[cases(%)].

Clinical data	Pre-PSM		
	Ki67 high expression(n=83)	Ki67 low expression(n=97)	P
Age(>55)	41(49.4)	45(46.4)	0.687
Hepatitis B history	76(91.6)	83(85.6)	0.211
Liver cirrhosis	58(69.9)	56(64.4)	0.092
Pre-operative liver function(A)	82(98.8)	94(96.9)	0.392
AFP(>200 μg/liter)	29(34.9)	25(25.8)	0.181
Diameter of tumor (Poorly differentiated, undifferentiated)	28(33.7)	25(25.8)	0.243
MVI(>M1)	29(34.9)	35(36.1)	0.873
Number of lesions(single)	69(83.1)	79(81.4)	0.768
Maximum diameter of lesions (>5 cm)	45(54.2)	49(50.5)	0.620

There was no statistical difference in the covariates.

We divided the 180 patients with HCC into 97 patients with low expression of Ki67 and 83 patients with high expression of Ki67 [a Ki67 < 20% was considered low expression and a Ki67 ≥ 20% was considered high expression (12)]. The two groups of patients were further divided into surgery and surgery + TACE sub-groups. In the Ki67 low expression group, the proportion of patients >55 years of age in the pre-PSM subgroup was higher than the surgery + TACE subgroup. After propensity score matching eliminated the different variables, 36 pairs of liver cancer patients were created. There was no statistical difference between the two covariates (P value >0.05, **Table 3**). In the Ki67 high expression group, there was no statistical difference in the covariates between the surgery and surgery + TACE subgroups (P value >0.05, **Table 4**). In all surgery + TACE patients, there was no statistical difference in the covariates between the Ki67 high- and low-expression subgroups (P value >0.05, **Table 5**).

**Table 3:** Basic information of the Ki67 low-expression group[cases(%)].

Clinical data	Pre-PSM			After PSM		
	Operating group (n=51)	operation + TACEgroup (n=46)	P	Operating group (n=36)	operation + TACEgroup (n=36)	P
Age(>55)	29(56.9)	16(34.8)	0.029	15(41.7)	13(36.1)	0.629
Gender(male)	42(82.4)	38(82.6)	0.974	30(83.3)	30(83.3)	1.000
Hepatitis B history	41(80.4)	42(91.3)	0.127	32(88.9)	33(91.2)	0.691
liver cirrhosis	34(66.7)	22(47.8)	0.061	20(55.6)	17(47.2)	0.479
Preoperative liver function(A)	51(100.0)	43(93.5)	0.064	36(100.0)	36(100.0)	1.000
AFP(>200 μg/liter)	13(25.5)	12(26.1)	0.947	9(25.0)	10(27.8)	0.789
Number of lesions(single)	44(86.3)	35(76.1)	0.197	29(80.1)	28(77.8)	0.772

The proportion of patients >55 years of age in the pre-PSM subgroup was higher than the surgery + TACE subgroup.

**Table 4:** Basic information of the Ki67 high expression subgroup[cases(%)].

Clinical data	Pre-PSM		
	Operating group(n=43)	operation + TACEGroup(n=40)	P
Age(>55)	22(51.2)	16(40.0)	0.739
Gender (male)	41(95.3)	36(90.0)	0.347
Hepatitis B history	40(93.0)	36(90.0)	0.620
Liver cirrhosis	32(74.4)	26(65.0)	0.350
Preoperative liver function(A)	42(97.7)	40(100.0)	0.332
AFP(>200 μg/liter)	15(34.9)	14(35.0)	0.991
Number of lesions(single)	38(88.4)	31(77.5)	0.186

There was no statistical difference in the covariates.

**Table 5:** Basic information of the operation + TACE group[cases(%)].

Clinical data	Pre-PSM		
	Ki67 high expression(n=40)	Ki67 low expression(n=46)	P
Age(>55)	19(47.5)	16(34.8)	0.231
Hepatitis B history	36(90.0)	42(91.3)	0.835
Liver cirrhosis	26(65.0)	22(47.8)	0.110
Preoperative liver function(A)	40(100.0)	43(93.5)	0.100
AFP(>200 μg/liter)	14(35.0)	12(26.1)	0.369
Diameter of tumor (Poorly differentiated, undifferentiated)	15(37.5)	10(21.7)	0.108
MVI(>M1)	29(72.5)	35(76.1)	0.704
Number of lesions(single)	31(77.5)	35(76.1)	0.877
Maximum diameter of lesions (>5 cm)	22(55.0)	22(47.8)	0.507

There was no statistical difference in the covariates.

### Effect of PA-TACE on Tumor Recurrence and OS in HCC Patients

**Figure 1:**
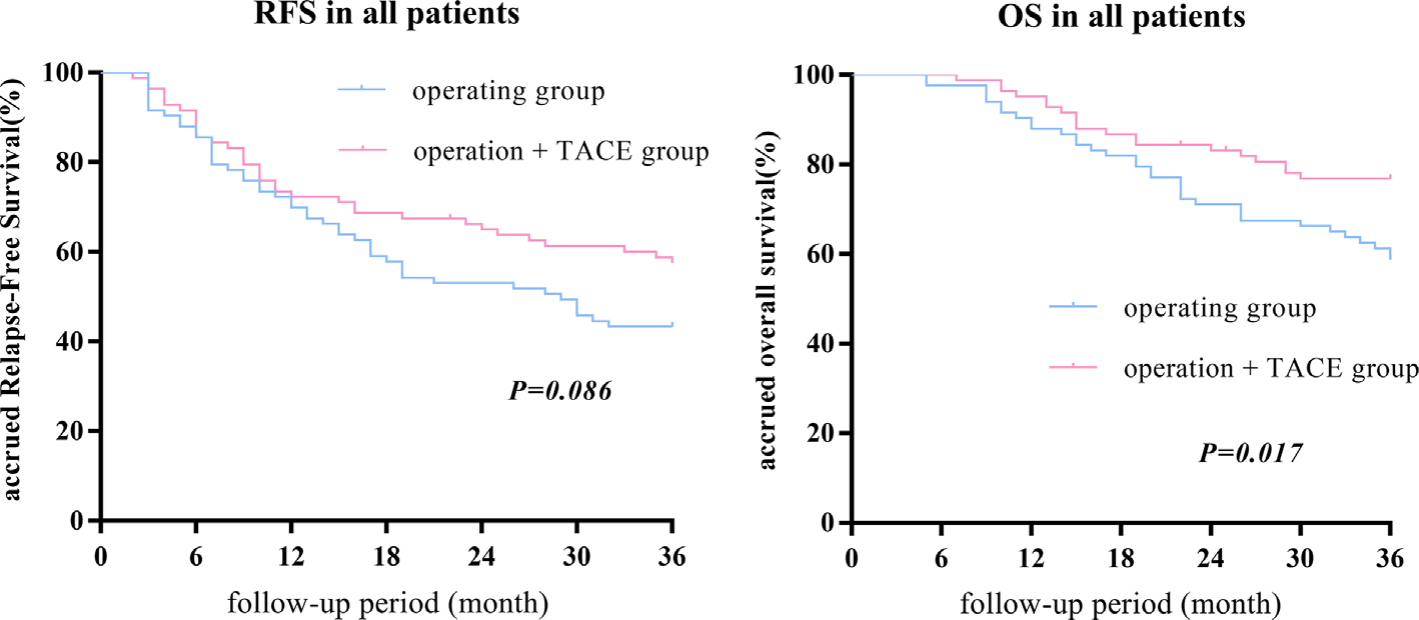
After PSM, there was no statistical difference between the 1-, 2-, or 3-year RFS between the surgery + TACE and surgery subgroups, while the OS in the surgery + TACE subgroup was significantly higher than the surgery subgroup.

After PSM, there was no statistical difference in the 1-, 2-, or 3-year RFS between the surgery + TACE and surgery subgroups, while the OS in the surgery + TACE subgroup was significantly higher than the surgery subgroup (RFS: 72.3%, 64.6%, and 56.8% vs. 69.9%, 53.0%, and 43.4%, respectively, P = 0.086; OS: 95.2%, 82.9%, and 76.5% vs. 88.0%, 71.1%, and 58.0%, respectively, P = 0.017; **Figure 1**).

### Effect of PA-TACE on Tumor Recurrence and OS in the Ki67 Low Expression Group

**Figure 2:**
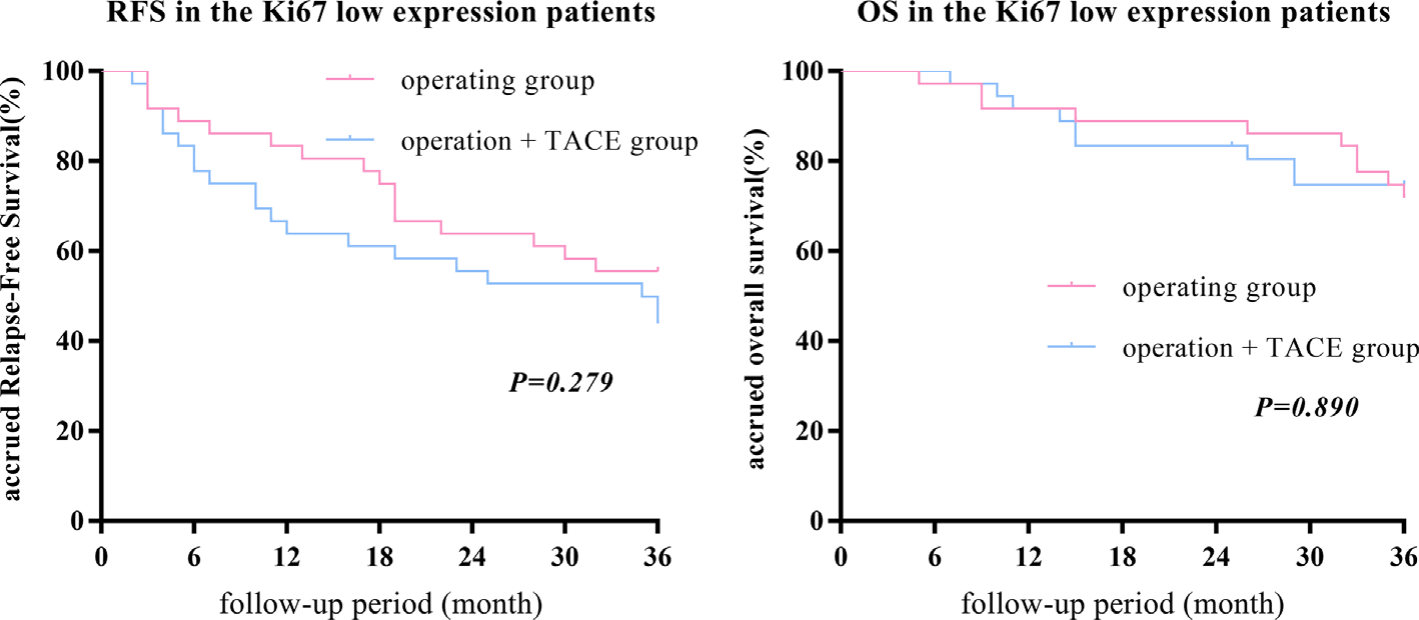
After PSM, there was no statistically significant difference in the 1-, 2-, or 3-year RFS and OS between the surgery + TACE and surgery subgroups in the Ki67 low-expression group.

After PSM, there was no statistically significant difference in the 1-, 2-, or 3-year RFS and OS between the surgery + TACE and surgery subgroups (RFS: 63.9%, 55.6%, and 42.9% vs. 83.3%, 63.9%, and 55.6%, respectively, P = 0.279; OS: 91.7%, 83.3%, and 74.3% vs. 91.7%, 88.9%, and 71.4%, respectively, P = 0.890; **Figure 2**).

### Effect of PA-TACE on Tumor Recurrence and OS in the Ki67 High Expression Group

**Figure 3:**
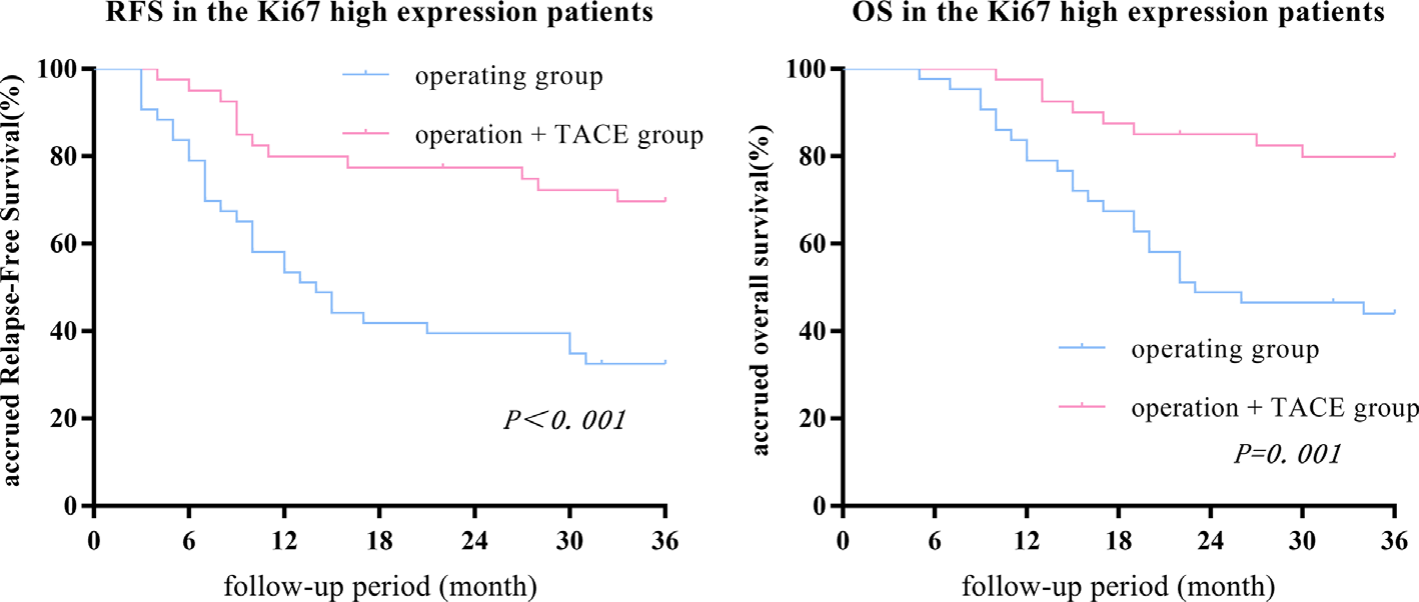
The 1-, 2-, and 3-year RFS and OS in the surgery + TACE subgroup were higher than the surgery subgroup in the Ki67 high-expression group. The differences in RFS and OS between the two groups were statistically significant.

The 1-, 2-, and 3-year RFS and OS in the surgery + TACE subgroup were higher than the surgery subgroup (RFS: 80.0%, 77.5%, and 69.2% vs. 53.5%, 39.5%, and 32.6%, respectively, P<0.001; OS: 97.5%, 85.0%, and 79.5% vs. 79.1%, 48.8%, and 42.9%, respectively, P = 0.001). The differences in RFS and OS between the two groups were statistically significant (**Figure 3**).

### Effect of PA-TACE on Tumor Recurrence and OS in the Surgery + TACE Group

**Figure 4:**
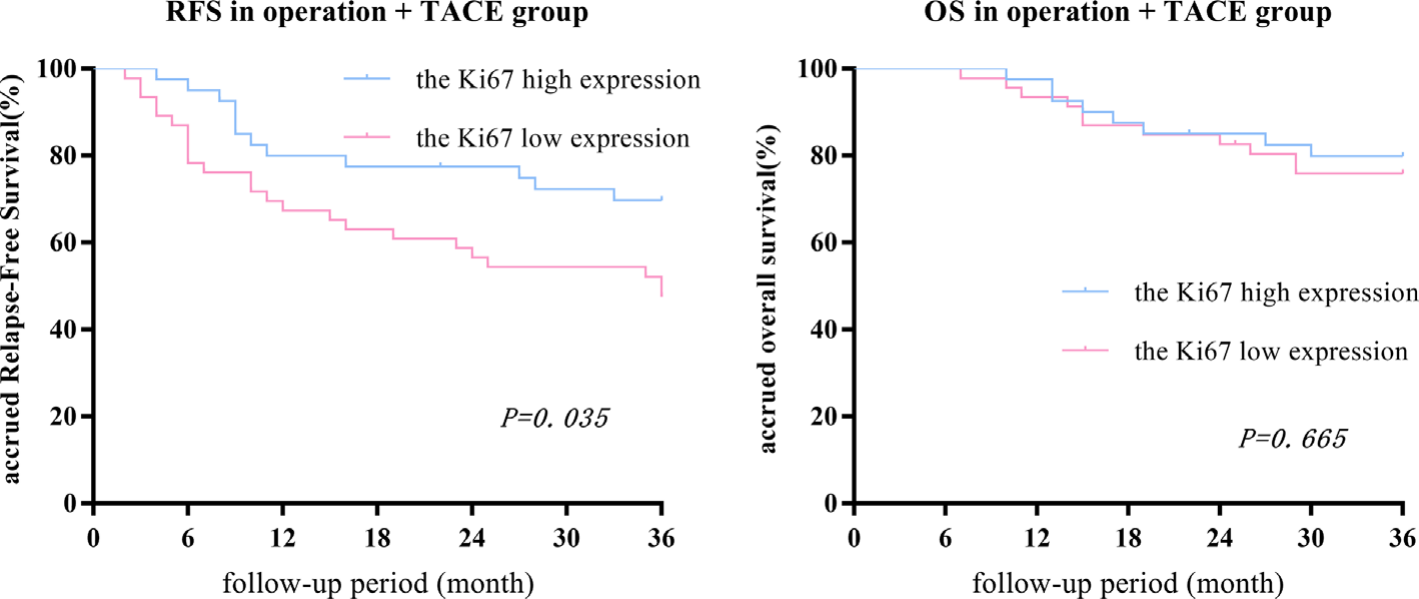
In the surgery + TACE group, there was no statistical difference between the 1-, 2-, or 3-year OS between the Ki67 low- and high-expression subgroups, while the RFS in the high-expression subgroup was significantly higher than the low-expression subgroup.

After PSM, there was no statistical difference in the 1-, 2-, or 3-year OS between low- and high-expression subgroups, while the RFS in the high-expression subgroup was significantly higher than the low-expression subgroup (RFS: 80.0%, 79.5%, and 69.2% vs. 67.4%, 56.5%, and 46.7%, respectively, P = 0.035; OS: 97.5%, 85.0%, and 79.5% vs. 93.5%, 82.6%, and 75.6%, respectively, P = 0.665; **Figure 4**).

### Multivariate Analysis on Recurrence and Survival of Patients With HCC After Surgery

After PSM, multivariate analysis in the Ki67 low expression group showed that a preoperative AFP level >200μg/liter (13) and a tumor maximum diameter ≥5 cm (14) were independent risk factors for RFS and OS rates in patients post-operatively, and liver cirrhosis was an independent risk factor for RFS (HR = 3.50 and 2.58, P = 0.001 and 0.015, respectively; and HR = 4.10 and 2.79, P = 0.006 and 0.041, respectively; and HR = 2.39, P = 0.02). Multivariate analysis in the Ki67 high expression group showed that postoperative adjuvant TACE treatment was an independent protective factor for RFS and OS after surgery (HR = 0.27, P < 0.001 and HR = 0.21, P < 0.001, respectively; **Table 6**).

**Table 6:** Multivariate analysis on recurrence and survival of patients with HCC after surgery.

Factors	the Ki67 low-expression subgroup				the Ki67 high-expression subgroup			
	RFS		OS		RFS		OS	
	HR(95%CI)	P	HR(95%CI)	P	HR(95%CI)	P	HR(95%CI)	P
Hepatitis B history	−	−	−	−	−	−	−	−
Liver cirrhosis	2.39(1.15–4.95)	0.02	−	−	−	−	−	−
AFP(>200 μg/liter)	3.50(1.62–7.56)	0.001	4.10(1.49–11.3)	0.06	−	−	−	−
Number of lesions(multiple)	−	−	−	−	−	−	−	−
Maximum diameter of lesions (>5 cm)	2.58(1.20–5.54)	0.015	2.79(1.04–7.43)	0.041	−	−	−	−
PA-TACE	−	−	−	−	0.27(0.13–0.55)	<0.001	0.21(0.89–0.49)	<0.001

## Discussion

HCC is the third most common cause of cancer-related deaths worldwide (15). If a radical liver resection is performed on patients with HCC, the 5-year recurrence rate is approximately 70%. The 5-year survival rate of patients with recurrent HCC decreases by 24% compared to patients without relapse, and the median survival time decreases by 54 months, which has a significant effect on prognosis (2). Postoperative adjuvant treatments, such as TACE, targeted therapy, and immunotherapy, can effectively delay tumor recurrence and prolong patient survival. Among the postoperative adjuvant treatments, PA-TACE is currently the most widely used (3).

The curative effects of PA-TACE are as follows: 1) TACE can increase the local drug concentration, which inhibits tumor proliferation and reduces the probability of recurrence (15); and 2) residual liver postoperatively can trigger the rapid regeneration phase, and proliferation of the residual tumor cells is apparent, which is more susceptible to chemotherapy drugs. Ki67 is an antigen related to cell proliferation, thus reflecting the activity of cell proliferation (5). Therefore, we speculated that HCC patients with high expression of Ki67 have more active cell proliferation, and the effect of adjuvant TACE after surgery will be superior. There are very few studies, however, involving the relationship between Ki67 and PA-TACE (11).

This study conducted a retrospective analysis involving 180 patients with HCC who underwent radical liver resections. There was no significant difference in the 1-, 2-, and 3-year RFS between the surgery + TACE and surgery subgroups, while the OS of the surgery + TACE subgroup were significantly greater compared with the surgery subgroup, which is in agreement with previous studies (16–18). A further stratified subgroup analysis showed that in the Ki67 low expression group, there was no significant difference in the 1-, 2-, and 3-year RFS and OS between the surgery + TACE and surgery subgroup. In the Ki67 high expression group, the 1-, 2-, and 3-year RFS and OS of the surgery + TACE subgroups were greater than the surgery group. The RFS was higher in the Ki67 high-than low-expression subgroup after 1, 2, and 3 years, and OS was no significant difference in all surgery + TACE patients. Multivariate analysis in the Ki67 high expression group showed that postoperative adjuvant TACE treatment was an independent protective factor for postoperative RFS and OS postoperatively.

Although TACE is currently widely used in the treatment of HCC, the indications for postoperative adjuvant TACE treatment are still controversial. Multi-center studies have shown that postoperative adjuvant TACE therapy can significantly improve the RFS but does not significantly improve the OS. Further stratification studies have shown that for patients at high risk for a post-operative recurrence with tumors >5 cm in diameter, MVI-positive tumors, and poorly differentiated HCC, PA-TACE can significantly improve the prognosis of patients (4). Studies have also shown that postoperative adjuvant TACE have shown a survival rate advantage in the case of vascular invasion or large HCC (diameter >5 cm) (19). In addition, studies have suggested that postoperative adjuvant TACE has a positive effect on patients with HCC that are prone to early recurrence and can be used as an empirical active intervention measure to prevent tumor recurrence (20). Studies have shown that PA-TACE can prolong the OS and DFS of MVI-positive patients, but is unrelated to the OS and DFS of MVI-negative patients (21). The current study showed that the RFS and OS in the surgery + TACE subgroup in the Ki67 high expression group were greater than the surgery subgroup, while there was no statistical difference between the two subgroups in the Ki67 low expression group, suggesting that the tumor Ki67 level can be used as a criterion for performing PA-TACE after radical liver tumor resection and screen suitable patients for effective anti-relapse treatment. There was no statistical difference between the Ki67 low- and high-expression subgroups at high risk for recurrence (number of lesions, maximum diameter of lesions, MVI,and the degree of tumor differentiation). This finding suggests that the tumor Ki67 level is an independent influencing factor unrelated to these factors. In addition, there was no statistical difference in the OS between the Ki67 low- and high-expression subgroups in all surgery + TACE patients, while the RFS in the high- was significantly greater than the low-expression subgroup. There was a greater balance between the two subgroups in terms of risk factors for recurrence. Ki67 is internationally recognized as the tumor proliferation index, and the higher the value, the worse the prognosis, which is in contrast to our results. This discrepancy may be related to the line of PA-TACE, which also demonstrates our conclusion that patients with high Ki67 expression have survival benefit from PA-TACE.

Studies have shown that After TACE treatment, the tumor Ki67 level and percentage of necrotic cells in tumor tissues were significantly higher than before treatment, and the level of Ki67 was positively correlated with the percentage of necrosis (22, 23), which is in agreement with our conclusions; however, we showed that some patients exist with high expression of Ki67 and non-poorly differentiated tumors who have a better prognosis after adjuvant TACE treatment. This finding suggests that post-operative TACE treatment based on the Ki67 level may be more accurate, and further stratified analysis needs to be carried out. The nuclear Ki-67 protein is related to cell proliferation activity and highly expressed in the G2/M phase, while physical factors, such as hypoxia and lack of a blood supply, have a greater impact on cells with vigorous replication in the G2/M phase (22), which may explain why TACE is more effective in patients with high Ki67 expression. In addition, the pharmacologic effects of chemotherapeutic drugs, such as doxorubicin, enter the nucleus to interfere with the transcription process, prevent mRNA synthesis, block the cell cycle, and inhibit tumor growth in the G2 phase of cell division (24), which is more effective in the Ki67 high expression group.

The current study showed that the 1-, 2-, and 3-year RFS and OS of the surgery + TACE subgroup in the Ki67 low expression group were less than the surgery subgroup. This may be related to the activation of related signaling pathways after embolization and the induction of neoangiogenesis, forming a microenvironment conducive to tumor proliferation (25). In addition, the serum concentrations of vascular endothelial and fibroblast growth factors increase after TACE, and the serum levels are positively correlated with tumor progression (26). Tumor cells with low Ki67 expression are less sensitive to chemotherapeutic drugs, and the therapeutic effect of the drugs is less than the proliferation induced by hypoxia. As a result, patients with low Ki67 expression will have a worse prognosis after TACE treatment; however, there is no statistical difference between the two sub-groups, which may be related to the small sample size. Thus, a corollary with a larger sample size is warranted. Studies have shown that the tumor Ki67 is not static. After TACE treatment, the expression of Ki67 in HCC tissues is higher than untreated HCC tissues, which may be related to the cell cycle of tumor induced by local treatment (27). This finding also indicates that TACE treatment in patients with low expression of Ki67 may lead to a poor prognosis.

This study had several limitations. First, our clinical data only represented a single research center, the sample size was small, and the conclusion was based on a retrospective analysis. Thus, a prospective, multi-center, large-sample random clinical trial should be conducted to further verify the relationship between Ki67 expression and PA-TACE with survival benefits. Second, the results of this study were from East Asia, and most of the patients had HCC caused by hepatitis B, which may not be applicable to hepatitis C or alcohol-related HCC. Third, the medications used in all TACE treatments in the current study were basically the same, and whether different medications led to different survival benefits remains to be further studied. Fourth, the current study was stratified according to a Ki67 ≥20% and <20%, but other studies have shown different stratification parameters (28); the precise cut-off value needs to be established. In addition, the prognosis of patients with early post-operative recurrence was extremely poor, which may represent the true recurrence of the primary cancer spread *via* the portal vein before HCC resection (2). The extensive clinical application of immunotherapy and targeted therapy in recent years has prolonged the survival time of patients with distant metastases of HCC after a recurrence; however, the time span of data collection in our center was relatively long. These factors may have an impact on the conclusions in the current study.

This study analyzed the relationship between the expression of Ki67 and the efficacy of postoperative adjuvant TACE in patients with HCC, and confirmed that when Ki67 was highly expressed, adjuvant TACE after liver resection could effectively reduce postoperative tumor recurrence and significantly improve the long-term survival rate, while when the expression of Ki67 was low, PA-TACE may result in a poor prognosis. Therefore, for HCC patients with high expression of Ki67, TACE treatment is recommended to improve the prognosis after liver resection.

## Data Availability Statement

The raw data supporting the conclusions of this article will be made available by the authors, without undue reservation.

## Ethics Statement

The studies involving human participants were reviewed and approved by the Institutional Review Board of The First Affiliated Hospital of Chongqing Medical University. Written informed consent for participation was not required for this study in accordance with the national legislation and the institutional requirements.

## Author Contributions

Y-FZ performed the study and wrote the paper. WT, KC, and XY collected and analyzed the data and made the tables and figures. Z-RS and XX designed the study, edited the manuscript, and offered suggestions for this study. Y-FZ and XX contributed equally to this work. All authors contributed to the article and approved the submitted version.

## Funding 

*In vitro* high-throughput drug sensitivity screening with patient-derived primary cells as a guide for clinical practice in hepatocellular carcinoma (2019GDRC002); Differentiation of adipose mesenchymal stem cells into hepatocytes induced by HNF-4α combined with HNF-3γ (cstc2019jcyj-msxmX0837).

## Conflict of Interest

The authors declare that the research was conducted in the absence of any commercial or financial relationships that could be construed as a potential conflict of interest.

## Abbreviations

PA-TACE, post-operative adjuvant transcatheter arterial chemoembolization; PSM, propensity score matching; RFS, recurrence-free survival; OS, overall survival; TACE, transcatheter arterial chemoembolization; DFS, disease-free survival; HCC, hepatocellular carcinoma.
